# Improving oral health in migrant and underserved populations: evaluation of an interactive, community-based oral health education program in Washington state

**DOI:** 10.1186/s12903-019-0723-7

**Published:** 2019-02-13

**Authors:** Ileana Ponce-Gonzalez, Allen Cheadle, Gino Aisenberg, Laura Flores Cantrell

**Affiliations:** 1 Community Health Worker Coalition for Migrants & Refugees, Department of Health Services, University of Washington, Washington, USA; 2 Center for Community Health and Evaluation, Kaiser Permanente Washington Health Research Institute, 1730 Minor Ave, Suite 1600, Seattle, WA 98101 USA; 3 School of Social Work, University of Washington, Seattle, WA USA; 4 Andy Hill Cancer Research Endowment, Seattle, WA USA

**Keywords:** Community health workers, Oral health education, Migrant and underserved populations, Latino

## Abstract

**Objectives:**

Oral health is one of the greatest unmet health needs of migrant farmworkers and many migrant workers lack basic oral health knowledge. This paper presents evaluation results for an oral health education program designed to both increase knowledge concerning oral health practices and to gain a better understanding of the knowledge, attitudes and behaviors regarding oral health among migrant workers.

**Methods:**

We used a pre-post uncontrolled design to assess the impact of the education program on participant knowledge about oral health practices. Changes in knowledge were assessed using a paper and pencil survey given to participants before the session began (pre) and at the end of the session (post). The pre-post survey was supplemented by qualitative information in the form of participant self-reported barriers and facilitators, and figure drawings illustrating their feelings about the state of their own oral health.

**Results:**

There were 311 participants in 12 workshops held in 2017 throughout Washington State. There were statistically significant increases in knowledge for all of the pre/post survey questions. Questions with particularly large improvements included: the results of having a mouth infection, factors causing oral health problems, and whether children in low-income families experience more tooth decay.

**Conclusions:**

An interactive, lay-led oral health education program can be an effective way to increase oral health knowledge in migrant populations. Recommendations for similar programs include using interactive approaches to engage participants, being open to learning and changing your own thinking, and using lay leaders for the education sessions.

## Introduction

Oral health is one of the greatest unmet health needs of migrant farmworkers [1]. Poor oral health reduces quality of life and is related to systemic chronic conditions such as stroke, heart and lung disease, and diabetes [1, 2]. Migrant farmworkers experience 150 to 300% more decayed teeth than their peers and at least half of farmworker children have at least one and average of three teeth with cavities [3].

Migrant workers, most of whom are Spanish-speaking workers, face many barriers to receiving health care in general and dental health care in particular, including lack of transportation, insurance, and sick leave; the threat of wage or job loss; language barriers; lack of regular dental practitioner; and limited clinic hours [4]. In addition to these barriers in access, many migrant workers lack basic oral health knowledge, including the relationship between sweet foods and caries and the positive effects of good oral hygiene and fluoride on dental health and overall health [5].

Oral health training and education offers an important avenue to increasing knowledge about good oral health practices; see a recent review by Nakre et al. [6]. However, all of the 40 studies in the review evaluated education programs implemented in non-migrant populations that were led by dental professionals (dentists or dental hygienists). Migrant populations may be better reached by education programs led by community health workers and promotoras de salud, especially using an interactive approach [7].

This paper presents evaluation results for an oral health education program led by trained lay leaders and community health workers that uses an interactive approach to engage with participants. The goals of the program were to both increase knowledge concerning oral health practices and to gain a better understanding of the knowledge, attitudes and behaviors regarding oral health among migrant workers. We will present results related to both goals: [1] pre/post surveys documenting knowledge gained by participants, and [2] data gathered from participants on the barriers and facilitators to good oral health, and their own perspectives about their oral health gathered through figure drawings.

## Methods

### Program description

The oral health education program was a joint collaborative effort of the Community Health Worker Coalition for Migrants and Refugees (CHWCMR) and the Arcora Foundation. CHWCMR is a group of volunteers dedicated to the promotion, empowerment, leadership, continuing education and integration of Community Health Workers (CHWs) into the healthcare system to improve the quality of life of migrants, the mobile poor, and refugees. A community health worker is a frontline public health worker who is a trusted member of and/or has an unusually close understanding of the community served. This trusting relationship enables the worker to serve as a liaison/link/intermediary between health/social services and the community to facilitate access to services and improve the quality and cultural responsiveness of service delivery [8]. CHWCMR members include CHWs, Community Health Representatives, Community Health Advisers and other related titles, and representatives of academic, media, government and private institutions. Arcora Foundation (formerly Washington Dental Service Foundation) is a nonprofit dedicated to improving oral health and health equity by partnering with communities to prevent oral disease, transform health systems, and increase access to care.

The project began in 2015 with an initial focus on developing a training program for CHWs to incorporate oral health into their work with migrants and refugees. A training curriculum was designed and refined using two focus groups of CHWs in central Washington State. In 2016 the training was implemented with 125 community champions, most of them CHWs, and an informal evaluation showed that it was effective in increasing CHW knowledge of evidence-based oral health practices. As word spread about the CHW training in Washington State, organizations serving migrants and refugees asked whether it could be given directly to community members that they served. In 2017 the curriculum was modified for a broader community audience. Modifications included adding more refugee populations and languages beyond Latino (Spanish) including Cambodian (Khmer), Chinese (Mandarin), Filipino (Tagalog), Vietnamese (Vietnamese), African (Amharic, Somali), and others. It was also expanded to include topics of particular interest to the community such as expanded diabetes and oral health content, culturally relevant prenatal oral health information, and HIV/AIDS and oral health.

The components of the two-hour program and their desired objectives are shown in Table 1. The program includes both didactic and interactive components. The figure drawing exercise and the gallery where participants shared thoughts on the determinants of their oral health provided a way of better engaging participants and facilitated gaining insights into their own experiences around oral health. Both methods are described in more detail in the data sources section below.

**Table 1:** Elements of the Oral Health Education Sessions

Section	Description	Objective
Presentation topics	Oral Health Statistics Oral Health conditions Oral Health Care Social Determinants of Health Affecting Oral Health The relationship between oral health and chronic conditions The role of Community Health Workers in Oral Health What your dentist expects from a patient Resources and tools for oral health in your community Being an advocate for oral health	Present information in an accessible way about key topics in oral health
Figure drawing exercise	Prompt for drawing: How do you feel about your own oral cavity? If your oral health is good then: What do you notice about how other members of your family feel about their oral health?	Allow participants to express their feelings about their oral health and how it affects them
Gallery	Exercise eliciting participant opinions about key social and other determinants of oral health	Identify key barriers and facilitators of good oral health from a community perspective
Fish Tank	Exercise about how to effectively clean your mouth. Participants take turns cleaning their own teeth and getting advice	Provide hands-on training for good oral health practices

Each session was led by two people: [1] a master trainer with a Masters in Public Health or a Diabetes Self-Management Program [9] credential, and [2] a lay leader who had received training for 4 hrs in the oral health curriculum. Master trainers volunteered their time; lay leaders were paid a $200 stipend for each session. Participants were given a $25 stipend for attending. All of the workshops included in this paper were conducted in Spanish.

### Evaluation design

We used a pre-post uncontrolled design to assess the impact of the education program on participant knowledge about oral health practices. Changes in knowledge were assessed using a paper and pencil survey given to participants before the session began (pre) and at the end of the session (post). The pre-post survey was supplemented by qualitative information in the form of participant self-reported barriers and facilitators, and figure drawings illustrating their feelings about the state of their own oral health. The evaluation design and methods were reviewed and approved by the Kaiser Permanente Washington Research Institute Institutional Review Board.

### Data sources

#### Pre/post surveys

A two-page survey was created that included basic demographic information and a series of multiple choice and true/false questions related to oral health. Participants were asked to complete the pre survey before the session began. It was emphasized on both pre and post occasions that it was acceptable to have wrong answers, that they should just answer as best they could. An ID number was created using initials and month of birth in order to link the pre and post surveys in the analysis, and the pre surveys were collected by the session leaders. The post survey was completed at the end of the session, writing the same ID on the form.

#### Small group discussions of oral health determinants

As part of the program, participants were divided into small groups and asked to brainstorm factors that affected their oral health, including structural, social, community, and cultural factors, after trainers explained social determinants of health concepts. Participants’ responses were recorded on flip charts which were collected at the end of the session and transcribed.

#### Figure drawings

The figure drawings were intended as an “icebreaker” exercise, to facilitate disclosure of personal perspectives and experiences while recognizing that it may be easier to express personal feelings through drawings rather than words. The exercise was introduced by saying: “Good day, we are going to talk about oral and dental health today. Rather than introduce each other, you are going to write your name on the page given to you, then you are going to draw the first thought you have about your own oral and dental health and how you feel about your mouth and teeth.” Participants were given the option to draw a picture representation of themselves or someone who was a friend or family member. It was not always indicated if the picture was of themselves or someone else.

### Analysis

The pre/post surveys were checked for incomplete answers and other data issues and then scanned into a database. We conducted descriptive statistics and paired t-tests for the knowledge questions using Stata [10]. The small group information was entered into a database and coded for themes.

Individual drawings were examined by a trained clinician and researcher who used a qualitative thematic approach. This researcher provided descriptive words to reveal the content of each drawing. From this data, themes were then identified and analyzed.

## Results

There were 311 participants in 12 workshops held between January 28th to December 11th, 2017 throughout Washington State. A total of 278 respondents completed both pre and post surveys (89% response rate). Demographics of those completing the survey are shown in Table 2; most participants were female (72%), 42% were between 25 and 49 years old, 29% had less than a high school education (and 20% had at least some college), and 78% listed Mexico as their country of origin.

**Table 2:** Demographics of education program participants

	Percent
Number of respondents	278
Gender	
Female	72.1
Male	26.8
Other	1.1
Age	
< 18 years	2.2
18–24	33.9
25–49	42.0
50–64	17.1
> 65 years	4.8
Education	
No formal education	4.9
Elementary school	24.2
High school	50.4
Some college	12.1
College degree+	8.4
Country of origin	
Mexico	77.8
United States	12.8
El Salvador	5.3
Other Latin America	4.1

Pre-post survey results are shown in Table 3. All of the improvements in knowledge were statistically significant and increases in the percent answered correctly from pre to post ranged from 7 to 27%. Questions with particularly large improvements included: the results of having a mouth infection, factors causing oral health problems, and whether children in low-income families experience more tooth decay.

**Table 3:** Changes in knowledge pre/post the education sessions

Question	Percent answering correctly			
	Pre	Post	Difference	*P*-value
Multiple choice				
Dry mouth can cause…difficulty speaking	57.4%	71.7%	14.3%	< 0.01
A mouth infection… can result in very serious complications	37.5%	60.5%	23.0%	< 0.01
Oral health problems can be due to…a number of factors (e.g., lack of economic resources	46.6%	73.8%	27.2%	< 0.01
When I have a cavity…I visit the dentist or other provider	41.3%	52.4%	11.1%	< 0.01
True/False				
Children in low-income families experience more tooth decay (True)	64.6%	86.4%	21.8%	< 0.01
Drinking juice is good for teeth (False)	82.3%	88.8%	6.5%	0.05
People with dentures need to visit the dentist (True)	86.8%	97.0%	10.2%	< 0.01
Fluoride is a naturally occurring mineral that heals and strengthens teeth (True)	86.5%	94.8%	8.3%	< 0.01

Table 4 lists the issues/barriers that were identified in the small group discussions. The most often mentioned barrier to good oral health was access – including lack of insurance and high cost of services. Other frequently mentioned barriers were language, legal status, social/economic status, and fear and trust.

**Table 4:** Determinants of health mentioned by education program participants

Issue/barrier	# of mentions^1^	Examples of responses
Access	32	Lack of insurance, high cost of services, no access to dental services, no dentist in rural areas
Dental practices	17	Not brushing every day, do not use floss, lack of sealants, use needle to clean teeth
Language/legal status	16	Language barriers, lack of legal status, limited English
Social/economic status	16	Housing problems, limited education, unemployed, poverty
Fear/trust	15	Do not trust the dentist, fear of dental procedures, sounds of drills
Food behaviors	13	Drink too much soda, eating sweets, junk food, chewing gum all of the time
Cultural	12	Different food cultures, family beliefs, myths, use of home remedies
Transportation	10	Lack of transportation
Knowledge	8	No oral health education, lack of information about resources
Time/energy	8	Lack of time
Substance use	7	Alcohol consumption, use of drugs, smoking
Water quality	5	Lack of safe drinking water, poor quality of water
Stigma/appearance	4	Stigma of losing teeth, nice smile improves appearance, stigma of HIV

Notes: 1 – Number of times mentioned across all of the education sessions – out of ~ 200 total mentions

**Fig. 1:**
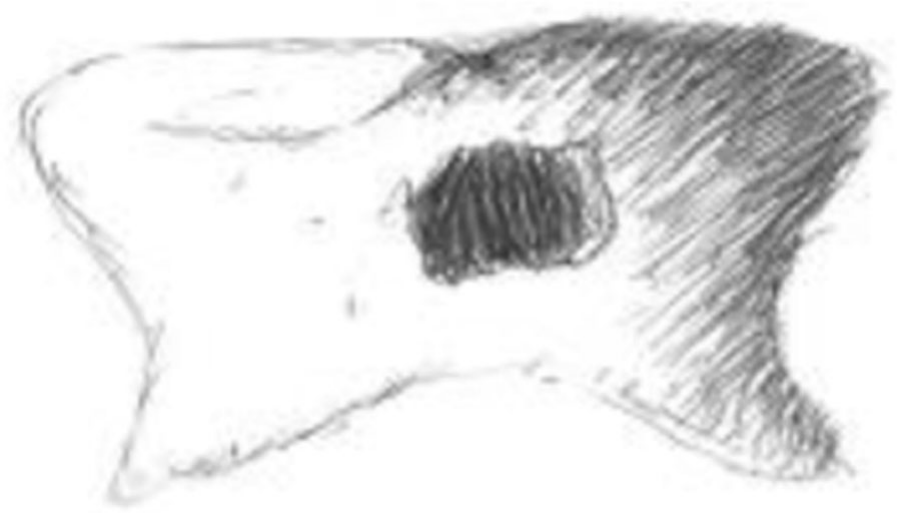
Sample participant figure drawing: Tooth decay on a single tooth

The examination of the figure drawings and the identification of their emerging themes provided crucial insights towards understanding the significant impact of oral health. Overall, the participants’ drawings depicted various stages of decay. While some respondents drew a single tooth (see Fig. 1), most drew a row or set of teeth. However, only a small number drew pictures that included a face, and of these drawings, only a few drew eyes, and lips and mouth revealing teeth. Some respondents included words with their drawings. Very few drawings depicted healthy teeth or a happy smile. In these instances, the large smiles signaling confidence in the appearance of their teeth which were uniformly properly aligned and shaped.

**Fig. 2:**
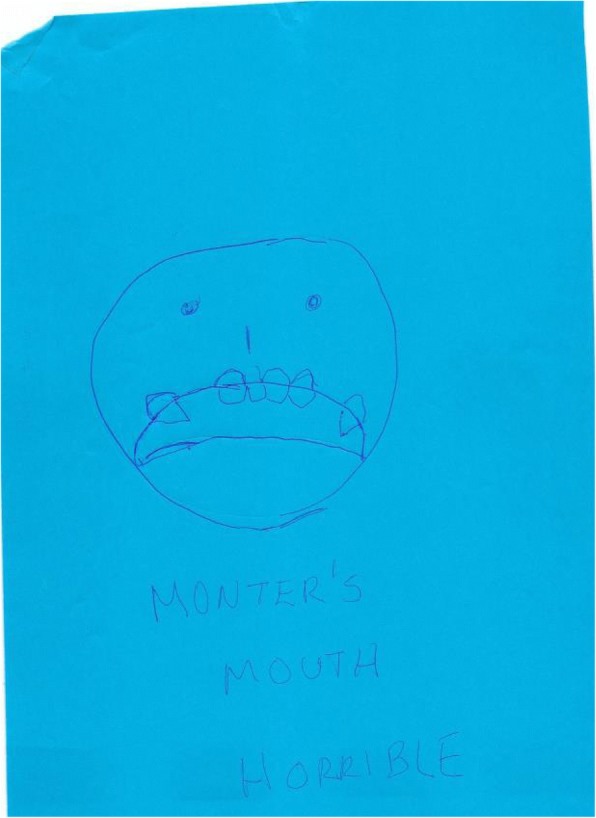
Sample participant figure drawing: Effect of bad teeth on self-esteem

The vast majority of drawings revealed poor condition of teeth and the serious consequences of delayed dental care. Advanced cavities were indicated by dense blackened areas of major portions of a tooth or several teeth. In some instances, respondents revealed the effects of delay of care by drawing missing teeth, and root and gum disease. In many instances, the drawings did not reveal a smile or a smile with teeth, signaling a sense of shame and a desire to hide the features and condition of their teeth. Figure 2 poignantly manifests the negative and debilitating impact on one’s sense of self and self-esteem.

Some respondents wrote words accompanying their drawings recognizing that the lack of dental insurance and the lack of money to pay for implants or other needed dental treatment were barriers to seeking care**.** For example**,** one respondent revealed that the lack of money and insurance are significant issues with regards to dental care. As a result, this person seeks care only when pain becomes too much. One man wrote; “Every 15 days I suffer and cry.” Another drew a picture of a broad shouldered male with tears streaming down his face and his hand holding a tooth and wrote the words, “Yes, men do cry.”

This delay in care due to financial constraints is a recurring theme that emerged from the drawings. Also, this delay in seeking care is a consequence of the lack of regular, preventative care experienced by most respondents. Unless their teeth or gums hurt, dental care is not often sought, even if the negative consequences are known. In one instance, a respondent delayed seeking care until pus had to be removed.

### Lessons learned

There are several lessons learned that were documented as part of the education sessions that may be helpful for others doing similar work.

#### Value of using lay leaders and an interactive approach

Lay leaders were better able to communicate with participants, translating oral health terms and concepts into language participants could understand. And having trained lay leaders increases community capacity to provide future trainings at relatively low cost. In addition, the small group discussions, figure drawings, and generally interactive approach to the sessions led to greater participant interest in engagement and likely contributed to the significant increases in knowledge that we found.

#### Insights from the figure drawings

There were a number of insights that the program designers gained, particularly through the figure drawings, that will be incorporated into future iterations of the program to improve outreach and education to this low-income population. These include [1] the development and refinement of health promotion materials and messaging related to prevention and children’s oral health; [2] messaging related to flossing and what is “normal” or a “warning sign” (e.g., bleeding gums); [3] health education related to risk factors, especially for children; and [4] health education regarding the use of emergency rooms.

#### Benefits of art and drawings

It was a significant benefit to incorporate the use of art and drawings in engaging and assessing Latinx individuals with regards to oral health. In addition, the results of this project highlight the importance of listening to, respecting and incorporating cultural norms. It is crucial that culturally inclusive and responsive practices be properly imbedded into provider trainings and overall oral health treatment of Latinx populations.

## Discussion

This paper presented evaluation results for an oral health education program designed to both increase knowledge concerning oral health practices and to gain a better understanding of the knowledge, attitudes and behaviors regarding oral health among migrant workers. There were statistically significant increases in knowledge for all of the pre/post survey questions; questions with particularly large improvements included: the results of having a mouth infection, factors causing oral health problems, and whether children in low-income families experience more tooth decay.

The most often mentioned barriers to good oral health mentioned in participant groups was access – including lack of insurance and high cost of services. Other frequently mentioned barriers were language, legal status, social/economic status, and fear and trust. The examination of the figure drawings and the identification of their emerging themes provided crucial insights towards understanding the significant impact of oral health. As the drawings poignantly revealed, many Latinx individuals experience diminished self-esteem and negative self-image as well as often endure emotional and physical pain. Overall, our positive findings for knowledge gain, and the high-levels of participant engagement in the group activities and drawings, support the idea that migrant populations may be better reached by education programs led by community health workers and promotoras de salud, especially those programs using an interactive approach [7].

There were several limitations that should be noted. The study used an uncontrolled design - we did not have a comparison group for the pre/post analysis. However, since the surveys were separated by only the training itself there were likely minimal history effects that a comparison group would have controlled for. More importantly, we were not able to conduct repeat surveys with more extended follow-up to see if the knowledge was retained beyond the day of the training. Finally, this was a community-based study that relied on the lay leaders to do the data collection. All of the lay leaders received training in how to message and administer the surveys but there may have been inconsistencies in survey administration, particularly the way the surveys were described to participants.

Despite the limitations, this study demonstrated that an interactive, lay-led oral health education program can be an effective way to increase oral health knowledge in migrant populations. Using community champions to train hard to reach populations seems to be a successful model for outreach, attendance and participation.

## Abbreviations

CHW: Community Health Worker
CHWCMR: Coalition for Migrants and Refugees
